# Semicarbazide-Sensitive Amine Oxidase (SSAO) and Lysyl Oxidase (LOX) Association in Rat Aortic Vascular Smooth Muscle Cells

**DOI:** 10.3390/biom12111563

**Published:** 2022-10-26

**Authors:** Vesna Manasieva, Shori Thakur, Lisa A. Lione, Jessal Patel, Anwar Baydoun, John Skamarauskas

**Affiliations:** 1 Imperial Biomedical Research Centre, Hammersmith Hospital, Department of Metabolism, Digestion and Reproduction, School of Medicine, Imperial College, London W12 0NN, UK; 2 Centre for Health Services and Clinical Research, Basic and Clinical Science Unit, Department of Clinical, Pharmaceutical and Biological Science, School of Life and Medical Sciences, University of Hertfordshire, Hatfield AL10 9AB, UK; 3 Leicester Institute of Pharmaceutical Innovation for Integrated Care (LIPIIC), Health and Life Sciences, De Montfort University, The Gateway, Leicester LE1 9BH, UK

**Keywords:** semicarbazide sensitive amine oxidase (SSAO), lysyl oxidase (LOX), copper amine oxidases, vascular smooth muscle cells, MDL72527, βAPN, benzylamine, cadaverine, vascular adhesion protein (VAP-1), amine oxidase copper containing 3 (Aoc3)

## Abstract

Vascular smooth muscle cells (VSMCs) are the main stromal cells in the medial layer of the vascular wall. These cells produce the extracellular matrix (ECM) and are involved in many pathological changes in the vascular wall. Semicarbazide-sensitive amine oxidase (SSAO) and lysyl oxidase (LOX) are vascular enzymes associated with the development of atherosclerosis. In the vascular smooth muscle cells, increased SSAO activity elevates reactive oxygen species (ROS) and induces VSMCs death; increased LOX induces chemotaxis through hydrogen peroxide dependent mechanisms; and decreased LOX contributes to endothelial dysfunction. This study investigates the relationship between SSAO and LOX in VSMCs by studying their activity, protein, and mRNA levels during VSMCs passaging and after silencing the LOX gene, while using their respective substrates and inhibitors. At the basal level, LOX activity decreased with passage and its protein expression was maintained between passages. βAPN abolished LOX activity (** *p* < 0.01 for 8 vs. 3 and * *p* < 0.05 for 5 vs. 8) and had no effect on LOX protein and mRNA levels. MDL72527 reduced LOX activity at passage 3 and 5 (^##^ *p* < 0.01) and had no effect on LOX protein, and mRNA expression. At the basal level, SSAO activity also decreased with passage, and its protein expression was maintained between passages. MDL72527 abolished SSAO activity (**** *p* < 0.0001 for 8 vs. 3 and * *p* < 0.05 for 5 vs. 8), VAP-1 expression at passage 5 (** *p* < 0.01) and 8 (**** *p* < 0.0001), and Aoc3 mRNA levels at passage 8 (* *p* < 0.05). βAPN inhibited SSAO activity (**** *p* < 0.0001 for 5 vs. 3 and 8 vs. 3 and * *p* < 0.05 for 5 vs. 8), VAP-1 expression at passage 3 (* *p* < 0.05), and Aoc3 mRNA levels at passage 3 (* *p* < 0.05). Knockdown of the LOX gene (**** *p* < 0.0001 for Si6 vs. Sictrl and *** *p* < 0.001 for Si8 vs. Sictrl) and LOX protein (** *p* < 0.01 for Si6 and Si8 vs. Sictrl) in VSMCs at passage 3 resulted in a reduction in Aoc3 mRNA (^####^ *p* < 0.0001 for Si6 vs. Sictrl and ^###^ *p* < 0.001 for Si8 vs. Sictrl) and VAP-1 protein (^#^ *p* < 0.05 for Si8 vs. Sictrl). These novel findings demonstrate a passage dependent decrease in LOX activity and increase in SSAO activity in rat aortic VSMCs and show an association between both enzymes in early passage rat aortic VSMCs, where LOX was identified as a regulator of SSAO activity, protein, and mRNA expression.

## 1. Introduction

Vascular smooth muscle cells (VSMCs) are the main stromal cells in the medial layer of the vascular wall, and are involved in many physiological functions and pathological changes that take place in the vasculature [1]. These cells produce the extracellular matrix (ECM) which is important for providing the arterial wall with the capacity to withstand the pressure from the circulating blood [1]. VSMCs are characterized as having high plasticity, which enables them to re-program their expression pattern in response to external stimuli. Therefore, based on the signals present in their local environment, these cells can change phenotype from a quiescent–contractile to a proliferative–synthetic phenotype. The acquired synthetic phenotype will enable these cells to migrate and proliferate inwardly from the medial to the intimal layer of the arteries, leading to intimal hyperplasia, which is one of the main characteristics in the pathophysiology of atherosclerosis [2].

Semicarbazide-sensitive amine oxidase (SSAO) is a vascular enzyme highly expressed in the VSMCs (localized in the caveolae of the plasma membrane) and endothelial cells (localized in the intracellular cytoplasmic vesicles). Being a copper amine oxidase, SSAO is encoded by the amine oxidase copper-containing 3 (Aoc3) gene, and distinguishes itself by its highly rich copper content [3]. SSAO exists as a soluble protein, and as a tissue bound type II transmembrane protein. The latter is often referred to as vascular adhesion protein-1 (VAP-1). SSAO catalyzes oxidative deamination of primary amines and converts them to aldehydes, with concomitant production of hydrogen peroxide (H_2_O_2_) and ammonia. In VSMCs, SSAO is important for vascular tone regulation, cell differentiation, and extracellular matrix organization [4,5,6]. Increased SSAO activity has been identified as a contributor in the development of atherosclerosis through the induction of cytotoxicity and elevated reactive oxygen species (ROS) levels in the vascular wall [7].

Lysyl oxidase (LOX) is another copper-containing amine oxidase which is abundantly present in the vascular smooth muscle cells, where it contributes to the formation and stability of the extracellular matrix (ECM). LOX oxidizes primary amine substrates, such as the epsilon amino group of lysine or hydroxylysine residues in collagens and lysine residues in elastin, and forms peptidyl α-aminoadipic-β-semialdehydes [8,9]. These highly reactive semi-aldehydes condense to form intra- and intermolecular covalent cross-linkages, which eventually lead to ECM formation and stability. Downregulated LOX has been associated with endothelial dysfunction [10,11,12] and upregulated LOX has been associated with induced VSMC migration, and solubilization of ECM components [10].

The very close similarity in terms of substrates and inhibitors poses a great challenge when studying SSAO and LOX activity in cell culture. Despite sharing common substrates and inhibitors, LOX is considerably smaller; its primary sequence lacks copper-coordinating histidines, and has lysine tyrosyl quinone (derived from the cross-linking of a modified tyrosine residue to the ε-group of a lysyl side chain) instead of topa-quinone (post-translationally modified tyrosine residue) as a cofactor [13]. Previous studies have already suggested a link between SSAO and LOX [4,14]; however, this has not been explored fully in a cell culture model. This study aims to identify a connection between these two enzymes in VSMCs, which could be a novel mechanism in the pathophysiology of atherosclerosis.

Here, we examined the activity, protein expression, and mRNA levels of SSAO and LOX in rat aortic VSMCs from passage 3, 5, and 8, using their respective substrates and inhibitors. SSAO and LOX activity were measured with the fluorometric Amplex^®^ Red Monoamine Oxidase assay, which has been previously implemented for the detection of LOX and SSAO-dependent H_2_O_2_ production [7,15,16,17]. This assay detects H_2_O_2_ in a horseradish peroxidase coupled reaction, using 10-acetyl-3,7-dihydroxyphenoxazine (Amplex^®^ Red reagent), a highly sensitive and stable probe for H_2_O_2_ [18]. Benzylamine was utilized as a model substrate to study SSAO activity, despite being also known as a substrate for LOX. This is because rodent SSAO has been demonstrated to possess a higher affinity for benzylamine compared to LOX, due to its narrower and more hydrophilic active site channel [19]. LOX activity was studied with 1,5-diaminopentane (cadaverine), due to its being extensively demonstrated as the most suitable substrate for an in vitro study of LOX activity [17,20,21].

MDL72527 was applied to irreversibly inhibit SSAO. MDL72527 is a propenyl amine-based inhibitor which was initially designed as polyamine oxidases (PO) inhibitor; however, it has also been identified as a mechanism-based, suicide inhibitor of SSAO [22,23,24]. Being a suicide inhibitor, MDL72527 is converted by SSAO to a highly reactive intermediate product. This leads to the irreversible inhibition of SSAO due to formation of a stable covalently bound enzyme-inhibitor adduct at the active site of the enzyme [25]. β-Aminopropionitrile (βAPN) was utilized to study LOX inhibition due to being reported as a highly potent mechanism-based inhibitor, with the strongest inhibitory potential for LOX in comparison to other copper amine oxidases present in the vasculature [4,10,26,27]. βAPN can also act as a weak and competitive reversible inhibitor for SSAO; however, in comparison to LOX, its inhibitory potential towards SSAO is less effective [4].

In this study, we provide evidence for association between SSAO and LOX in rat aortic VSMCs while demonstrating LOX as a main regulator of SSAO activity, VAP-1 protein, and Aoc3 mRNA expression in early passage rat aortic VSMCs.

## 2. Materials and Methods

### 2.1. Reagents

Cell culture reagents were purchased from Fisher Scientific (Loughborough, UK). Unless otherwise stated, chemicals and reagents were purchased from Sigma-Aldrich (Poole, UK).

### 2.2. Animals

Male Wistar rats (180–220 g) were housed in pairs in standard cages (Tecniplast 2000 P) with sawdust (Dates and grade 7 substrate) and shredded paper wool bedding with water and food (5LF2 10% protein LabDiet) in the Biological Services Unit at the University of Hertfordshire. The housing environment was maintained at a constant temperature (21 ± 2 °C) and light–dark cycle (12:12 h). All experiments were carried out in accordance with the University of Hertfordshire animal welfare ethical guidelines and European directive 2010/63/EU, and all tissues collected were naïve shared within teaching/research in accordance with the 3 Rs. Rats were euthanized by CO_2_ asphyxiation, and the aorta was removed and placed in a DMEM solution supplemented with 10% Fetal Bovine Serum (FBS (*v*/*v*)), 1% penicillin (100 units/mL), streptomycin (100 μg/mL), and 2 mM L-Glutamine.

### 2.3. Cells

The VSMCs were selected due to the fact that they expressed high levels of both SSAO and LOX. This study used primary VSMCs, because primary cell cultures most closely represent the tissue of origin. Primary cells require several passages to fully develop receptors and maintain a consistent and fast proliferation rate [28]; however, they can also lose the capacity to contract, and, therefore, transform into synthetic phenotypes in late passages [29]. These cells can prematurely undergo induction of cellular senescence earlier than other cells, causing early cultures to contain a mixture of both young and senescent cells [30]. VSMCs at early passage (passage 4) have shown better migration probability, and VSMCs at late passage (passage 10) have demonstrated a decrease in size and less elongated morphology, with losses in parallel organization of actin filament, and a randomized actin filament distribution [28]. Therefore, in order to follow changes in enzymatic activity, and investigate the association between SSAO and LOX, VSMCs at passages 3, 5, and 8 were used for experimental analysis.

### 2.4. Isolation and Characterization of Rat Aortic VSMCs

VSMCs were isolated from the rats’ aortas, as per standard protocol [29]. The rats were euthanized by CO_2_ asphyxiation followed by cervical dislocation. The aortas were removed and placed in a DMEM solution supplemented with 10% Fetal Bovine Serum (FBS (*v*/*v*)), 1% penicillin (100 units/mL), streptomycin (100 μg/mL), and 2 mM L-Glutamine. In order to characterize the cells, the isolated rat VSMCs were stained for the smooth muscle cell marker SM22α [31]. Please see Appendix A for isolated and characterized VSMCs images.

### 2.5. Amplex Red Assay

SSAO and LOX activity (expressed as H_2_O_2_ production) were studied with the Amplex^®^ Red assay [7,16,20]. Confluent rat aortic primary VSMCs from passages 3, 5, and 8 were treated with a reaction mixture containing 860 μL of 0.25 M sodium phosphate buffer (pH 7.4), 20 μL Amplex^®^ Red (20 mM), 10 μL horseradish peroxidase (200 U/mL), 10 μL clorgyline (0.5 mM), and 200 μL (0.5 mM) benzylamine, or 1,5 diamino pentane dihydrochloride (cadaverine), respectively. Clorgyline (MAO-A and MAO-B selective inhibitor) was added in the reaction mixture because benzylamine is a common substrate for not only SSAO and LOX, but also MAO-A and MAO-B. SSAO activity (expressed as H_2_O_2_ production) was also studied in the presence of MDL72527 and βAPN. Cells were treated with a reaction mixture containing 860 μL of 0.25 M sodium phosphate buffer (pH 7.4), 20 μL Amplex^®^ Red (20 mM), 10 μL horseradish peroxidase (200 U/mL), 10 μL clorgyline (0.5 mM), and 200 μL (0.5 mM) benzylamine, or 200 μL (0.5 mM) benzylamine and 10 μL (100 µM) MDL72527—irreversible SSAO inhibitor, or 200 μL (0.5 mM) benzylamine and 10 μL (200 µM) βAPN—irreversible LOX inhibitor.

LOX activity was assessed with cadaverine in the presence of MDL72527 and βAPN, respectively. Cells were treated with a reaction mixture containing 860 μL of 0.25 M sodium phosphate buffer (pH 7.4), 20 μL Amplex^®^ Red (20 mM), 10 μL horseradish peroxidase (200 U/mL), 10 μL clorgyline (0.5 mM), and 200 μL (0.5 mM) 1,5 diamino pentane dihydrochloride (cadaverine), or 200 μL (0.5 mM) cadaverine and 10 μL (100 µM) MDL72527, or 200 μL (0.5 mM) cadaverine and 10 μL (200 µM) βAPN. Cells containing reaction mixture without benzylamine or cadaverine were considered as controls. SSAO and LOX activity were measured after 6 h incubation with a reaction mixture at 37 °C, at excitation 540 nm and emission 590 nm, on a Clario Star^®^ Microplate Reader (BMG Labtech, Aylesbury, England). The end-point fluorescence was measured with resorufin standards after 1 h incubation at 37 °C. Then, 2 mM of resorufin stock solution was diluted to a concentration of 1000 μM in a 1X reaction buffer (2 mL of 5X reaction buffer (0.25 M sodium phosphate at pH 7.4), 10 mL distilled water) to yield resorufin standards ranging from 0 to 20 μM. The resorufin readings were used to prepare a standard curve of resorufin fluorescence (RFU) versus concentration (μM). In order to express SSAO/LOX activity in nmol H_2_O_2_/mL, the fluorescence readings from different time intervals were multiplied by the slope and added by the intercept (both calculated from the linear equation of the resorufin standard curve). In order to express SSAO/LOX activity in nmol H_2_O_2_/mg protein, the nmol H_2_O_2_/mL values were divided over the protein concentration (mg/mL), which was calculated with the BCA assay.

### 2.6. Western Blot Analysis

Protein was extracted from VSMCs cultured in control condition (non-treated), and after 1 h pre-treatment with 200 µM βAPN (previously established effective in inhibiting LOX [14,32] without inducing cytotoxic effects [14]), or 100 µM MDL72527 (demonstrated effective in inhibiting SSAO without causing cytotoxic effect on VSMCs—unpublished data) at passages 3, 5, and 8. Cell monolayers were lysed in an ice-cold radioimmunoprecipitation lysis buffer (50 mM tris hydrochloride, pH 7.4, 150 mM sodium chloride, 5 mM EDTA, and 0.1% sodium dodecyl sulphate [SDS]), with a protease and phosphatase inhibitor cocktail (Sigma-Aldrich, Poole, UK). Cell homogenates were sonicated for 5 min and centrifuged at 10,000 rpm at 4 °C before protein concentration determination using the Bradford assay. Loading samples were prepared using a Bolt lithium dodecyl sulfate sample buffer and a Bolt sample reducing agent (Life Technologies, Waltham, MA, USA), as per the manufacturer’s instructions. Samples were denatured at 70 °C for 10 min. Protein samples were loaded into Bolt Bis-Tris Plus Gels (8%) (Life Technologies) and run alongside a protein ladder (Precision Plus Protein Kaleidoscope Standard; Bio-Rad, Watford, UK). Gel electrophoresis was run at 160 V for 40 min in a Bolt MES SDS running buffer (Life Technologies) for low molecular weight proteins, or a MOPS SDS running buffer (Life Technologies) for high molecular weight proteins. Protein samples were subsequently transferred onto a nitrocellulose membrane using the iBlot 2 Transfer System (Life Technologies). Membranes were then blocked with 5% non-fat milk for 1 h, and subsequently incubated with the Alexa Fluor^®^ conjugated VAP-1 antibody (Santa Cruz, CA, USA, SC373924) at dilution 1:1000 in 5% *w*/*v* BSA, 1X TBS, 10% Tween^®^20, or rabbit monoclonal to Pro-LOX antibody (Abcam, Cambridge, UK), ab174316) at dilution 1:1000 in 5% *w*/*v* BSA, 1X TBS, 10% Tween^®^20, overnight at 4 °C. After washing, blots were incubated in horseradish peroxidase (HRP) conjugated secondary antibodies (Cell Signaling Technology, London, UK/Bio-Rad, Kidlington, UK) for 1 h at room temperature, apart from blots incubated with VAP-1. A β-actin-HRP-conjugated antibody (Abcam, ab207674) was used as a loading control, with blots incubated for 1 h at room temperature. A peroxidase detection system (Radiance ECL; Azure Biosystems, Dublin, UK) and myECL Imager (Thermo Fisher Scientific, Hemel Hempstead, UK) were used for the visualization of immunoreactivity. The protein bands were quantified with Image J software (National Institutes of Health and the Laboratory for Optical and Computational Instrumentation (LOCI, University of Wisconsin, Madison, WI, USA)

### 2.7. Quantitative Polymerase Chain Reaction (qPCR)

SSAO and LOX gene expression levels were detected and quantified with a quantitative polymerase chain reaction (qPCR) based on a relative quantification method, also known as comparative threshold method. The QPCRBIO SyGreen 1-Step Go Kit (PCR Biosystems, London, UK) was used to perform the PCR reaction. The primers for VAP-1, LOX, and β-actin (Table 1) were designed through Roche Life Sciences, by inserting their respective reference sequences (NM_017061.2 Rattus norvegicus lysyl oxidase (LOX), mRNA; NM_031582.2 Rattus norvegicus amine oxidase, copper containing 3 (Aoc3), mRNA), which were obtained from the ncbi.nlm.nih.gov website.

A master mix (with final volume 10 μL/well) was prepared for all reference (non-treated) and target (MDL72527 and βAPN treated) samples (Table 2). β-actin was used as a reference gene and Aoc3 and LOX were the genes of interest (target genes). Each target gene was run in triplicate, simultaneously with the reference gene β-actin, and was analyzed separately for all reference and target samples.

The master mix per well for each gene was prepared with: 1X qPCRBIO SyGreen 1-Step Mix (5 μL), forward primer at concentration 10 μM (0.4 μL), reverse primer at concentration 10 μM (0.4 μL), 1X RTaseGo—which contains RNase inhibitor (0.5 μL), 20 ng/μL template RNA (1 μL), and 2.7 μL qPCR grade water. Samples prepared without 1X RTaseGo were used as a negative control. After loading, the plate was centrifuged at 1000 RPM for 1 min, and readings were taken on the QuantStudio™ 7 Flex Real-Time PCR System (Thermo Fisher Scientific). The data were analyzed with the 2-ΔΔCT method of relative quantification [33].

### 2.8. siRNA Knockdown

Associations between LOX and SSAO in early passage rat aortic VSMCs were studied with siRNA gene knockdown using reverse transfection and lipofectamine—RNAiMAx transfection reagent [34]. In order to confirm a successful knockdown, HS cell death was used as a positive control due the fact that it contains a blend of a highly potent siRNAs, which target genes which are indispensable for cell survival [35]. It was prepared as a 10 μM solution by dissolving 1 nmol lyophilized siRNA with 100 μL RNase free water. The Si control was used as a negative control due to the fact that it is composed of random sequences that do not target any gene inside the cells [35]. It was prepared as a 20 μM solution by dissolving 5 nmol lyophilized siRNA with 250 μL RNase free water. In addition, a MOCK transfection control was also set up, which contained only transfection reagent and OptiMEM—a reduced serum medium that allows for a reduction in FBS supplementation of at least 50%, with no change in cell growth rate or morphology.

MOCK was included to observe any phenotypic changes to the cells caused by the transfection. Two siRNAs that target LOX gene, SiRNA_LOX_6 and SiRNA_LOX_8, were selected based on their target sequences (Table 3).

Separate T-75 flasks were used for transfection with individual siRNAs, Si control, and HS death. Four reaction mixtures were prepared with individual siRNAs, Si control, and HS death. In doing this, 8 μL RNAiMAX was added to 8 μL SiRNA_LOX_6 or SiRNA_LOX_8 at a concentration of 20 μM, and then supplemented with 1350 μL OptiMEM. In addition, 8 μL RNAiMAX was also added to 8 μL Si control (20 μM) or 16 μL HS cell death (10 μM), and then supplemented with 1350 μL OptiMEM. The reaction mixtures were incubated for 20 min at room temperature, during which early passage (2 and 3), fully confluent rat aortic VSMCs were harvested and resuspended in antibiotic-free DMEM (supplemented with 10% FBS) at a concentration of 3 × 10^5^ cells/mL. Then, 6.650 mL of cells were added to each T-75 flask, followed by the addition of the reaction mixture previously prepared, thus obtaining a final siRNA concentration of 20 nM and 2 × 10^6^ cells/flask. Cells were distributed evenly through the flask by swirling in a figure of eight, and the flasks were incubated at 37 °C/5% CO_2_. The following day, 6 mL of antibiotic-free DMEM (supplemented with 10% FBS) was added to each flask. When 72 h had passed post-transfection, the cells were harvested by trypsinization and re-suspended in two falcon tubes with 2 mL antibiotic-free DMEM (supplemented with 10% FBS) at a concentration of 5 × 10^4^ cells/mL. One falcon tube with cells was used for extraction of protein samples, and the other for the extraction of RNA. 

In order to extract protein samples, cells were first centrifuged at 2000 RCF for 6 min at 4 °C. The supernatant was removed, and cells were washed with 1X PBS and centrifuged again at 2000 RCF for 6 min at 4 °C. After the removal of the supernatant, the cells were re-suspended with 1mL RIPA buffer (0.5 M TRIS at pH 7.4, 0.9 g NaCl, 0.1 g SDS, 1 mL TRITON × 100, 5 mM EDTA), then supplemented with a 10% protease and phosphatase inhibitor cocktail. The samples were then moved to a centrifuge tube, vortexed for 30 s, sonicated in icy water for 5 min, and stored at −20 °C until further use.

In order to extract RNA, cells were centrifuged at 300 RCF for 5 min and re-suspended in 1× PBS, after which they were centrifuged again at 300 RCF for 5 min. The supernatant was then removed, and cells were re-suspended in 250 μL lysis buffer (prepared by adding 325 μL of 1-Thioglycerol to 32.5 mL of BL buffer, which was supplied with the ReliaPrep™ RNA cell miniprep system kit (Promega, Chilworth, UK). The lysate was mixed well by repeatedly pipetting 7–10 times, and was then transferred to a microcentrifuge tube. Afterward, 85 μL isopropanol (100%) was added to each centrifuge tube and the tubes were mixed by vortexing for 5 s.

The lysate from each centrifuge tube was then transferred to a ReliaPrep™ minicolumn, placed in a collection tube, and centrifuged at 13,000× *g* for 30 s at 22 °C. After centrifugation, the liquid was discarded and the minicolumn was placed in a different collection tube. A total of 500 μL of RNA wash solution (previously prepared by adding 60 mL of 95% ethanol to a 35 mL concentrated RNA wash solution—RWA) was added to the minicolumn, and the collection tube containing the minicolumn was centrifuged again at 13,000× *g* for 30 s. After that, 30 μL of DNase I incubation mix (24 μL yellow core buffer, 3 MnCl_2_ at 0.09 M, and 3 μL DNase I) was added to the minicolumn membrane, and the minicolumn was incubated for 15 min at 22 °C. After the incubation, 200 μL of column wash solution (prepared by adding 7.5 mL of 95% ethanol to a 5 mL concentrated column wash solution—CWE) was added to the minicolumn, and the minicolumn was centrifuged at 13,000× *g* for 15 s; after that, 500 μL RNA wash solution was added again, and the minicolumn was centrifuged at 13,000× *g* for 30 s. The wash solutions and the collection tube were then discarded. The minicolumn was placed to a new collection tube. Then, 300 μL of RNA wash solution was added again followed by centrifugation at 20,000× *g* for 2 min. The minicolumn was then transferred from the collection tube to an elution tube. Then, 30 μL of nuclease-free water was added to the minicolumn, and the elution tube containing the minicolumn was centrifuged at 13,000× *g* for 1 min. The purified RNA was quantified with a NanoDrop Spectrophotometer (NanoDrop Technologies, Wilmington, DE, USA) and stored at −80 °C until further use.

### 2.9. Statistical Analysis

The data was analyzed with the statistical software GraphPad Prism 7 (GraphPad, San Diego, CA, USA). Statistical comparisons were made using one-way or two-way ANOVA, followed by Dunnett’s or Tukey’s multiple comparison tests. Sidak’s multiple comparison test was applied for a set of means, to adjust significance level for multiple comparisons. Probability values < 0.05 were considered to be statistically significant.

## 3. Results

### 3.1. SSAO Activity in VSMCs from Passages 3, 5, and 8

**Figure 1:**
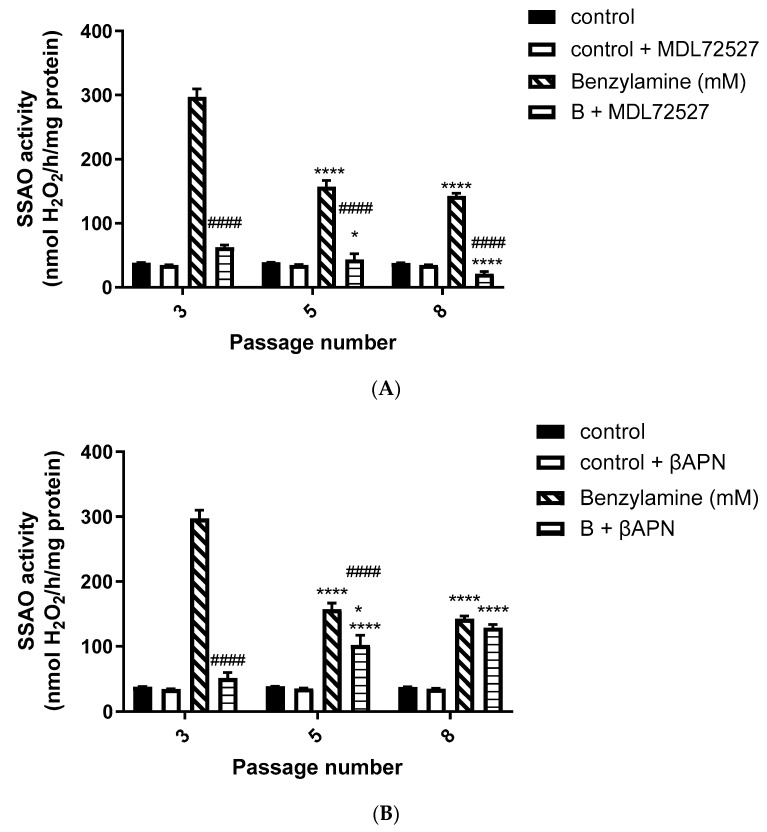
SSAO activity in VSMCs from passages 3, 5, and 8 at the basal level, and after MDL72527 and βAPN treatment. (**A**) Cells were treated with a reaction mixture containing 0.5 mM benzylamine (hatched bars); 0.5 mM benzylamine + 100 µM MDL72527 (horizontal line bars); a reaction mixture without benzylamine (control—solid bars) and a reaction mixture without benzylamine + MDL72527 (control + MDL72527, open bars).**** *p* < 0.0001 for cells passage 5 vs. 3 and 8 vs. 3 after treatment with benzylamine; **** *p* < 0.0001 for cells passage 8 vs. 3 and * *p* < 0.05 for cells passage 5 vs. 8 after treatment with benzylamine + MDL72527; ^####^ *p* < 0.0001 for B + MDL vs. benzylamine at passages 3, 5, and 8. (**B**) Cells were treated with a reaction mixture containing 0.5 mM benzylamine (hatched bars); 0.5 mM benzylamine + 200 µM βAPN (horizontal line bars); a reaction mixture without benzylamine (control—solid bars) and a reaction mixture without benzylamine + βAPN (control + βAPN, open bars).**** *p* < 0.0001 for cells passage 5 vs. 3 and 8 vs. 3 after treatment with benzylamine; **** *p* < 0.0001 for cells passage 5 vs. 3 and 8 vs. 3, and * *p* < 0.05 for cells passage 5 vs. 8 after treatment with benzylamine + βAPN; ^####^ *p* < 0.0001 for B + βAPN vs. benzylamine at passages 3 and 5. Values are mean ± S.E.M. (n = 5).

In order to investigate basal and enzyme-induced changes in SSAO activity (nmol H_2_O_2_/h/mg protein) during VSMCs passaging, SSAO activity was measured at the basal level using benzylamine as substrate (0.5 mM), and in the presence of SSAO and LOX respective irreversible inhibitors, MDL72527 and βAPN. Figure 1 shows the detected SSAO activity in benzylamine-treated cells, with and without the presence of MDL72527, and with and without the presence of βAPN.

### 3.2. LOX Activity in VSMCs from Passage 3, 5 and 8

**Figure 2:**
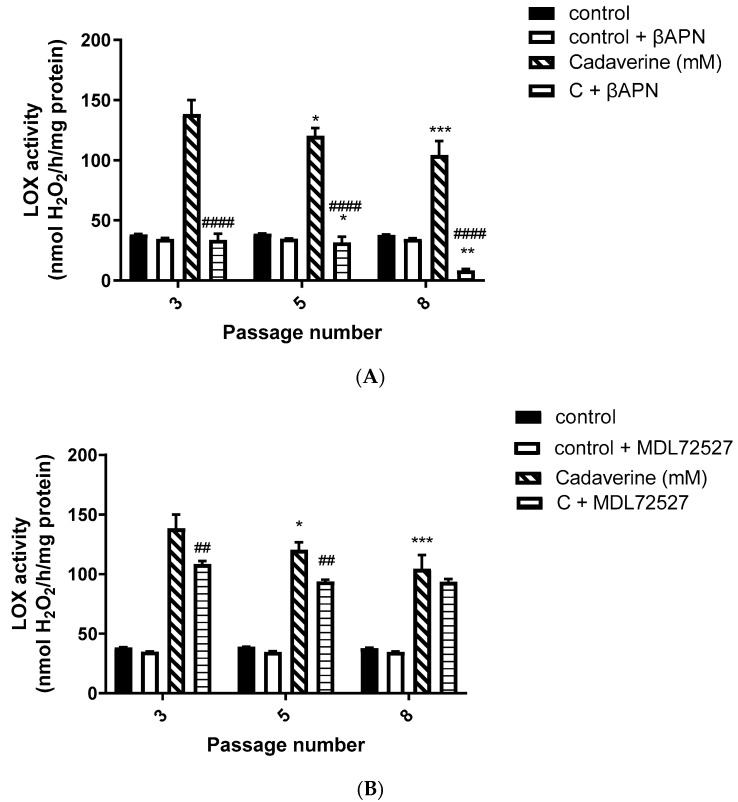
LOX activity in VSMCs from passages 3, 5, and 8 at the basal level, and after βAPN and MDL72527 treatment. (**A**) Cells were treated with a reaction mixture containing 0.5 mM cadaverine (hatched bars); 0.5 mM cadaverine + 200 µM βAPN (horizontal line bars); a reaction mixture without cadaverine (control—solid bars) and a reaction mixture without cadaverine + βAPN (control + βAPN, open bars). * *p* < 0.05 for cells passage 5 vs. 3 and *** *p* < 0.001 for cells passage 8 vs. 3 after treatment with cadaverine; ** *p* < 0.01 for cells passage 8 vs. 3 and * *p* < 0.05 for cells passage 5 vs. 8 after treatment with cadaverine + βAPN; ^####^ *p* < 0.0001 for C + βAPN vs. cadaverine at passages 3, 5, and 8. (**B**) Cells were treated with a reaction mixture containing 0.5 mM cadaverine (hatched bars); 0.5 mM cadaverine + 100 µM MDL72527 (horizontal line bars); a reaction mixture without cadaverine (control—solid bars) and a reaction mixture without cadaverine + MDL72527 (control + MDL72527, open bars).* *p* < 0.05 for cells passage 5 vs. 3 and *** *p* < 0.001 for cells passage 8 vs. 3 after treatment with cadaverine; ^##^ *p* < 0.01 for C + MDL72527 vs. cadaverine at passages 3 and 5. Values are mean ± S.E.M. (n = 5).

In order to investigate basal and enzyme-induced changes in LOX activity (nmol H_2_O_2_/h/mg protein) during VSMCs passaging, LOX activity was measured at the basal level using cadaverine as a substrate (0.5 mM), and in the presence of SSAO and LOX respective irreversible inhibitors, MDL72527 and βAPN. Figure 2 shows the detected LOX activity in cadaverine treated cells, with and without the presence of βAPN, and with and without the presence of MDL72527.

### 3.3. VAP-1 and LOX Protein Expression in VSMCs from Passage 3, 5 & 8 at Basal Level and after βAPN and MDL72527 Treatment

**Figure 3:**
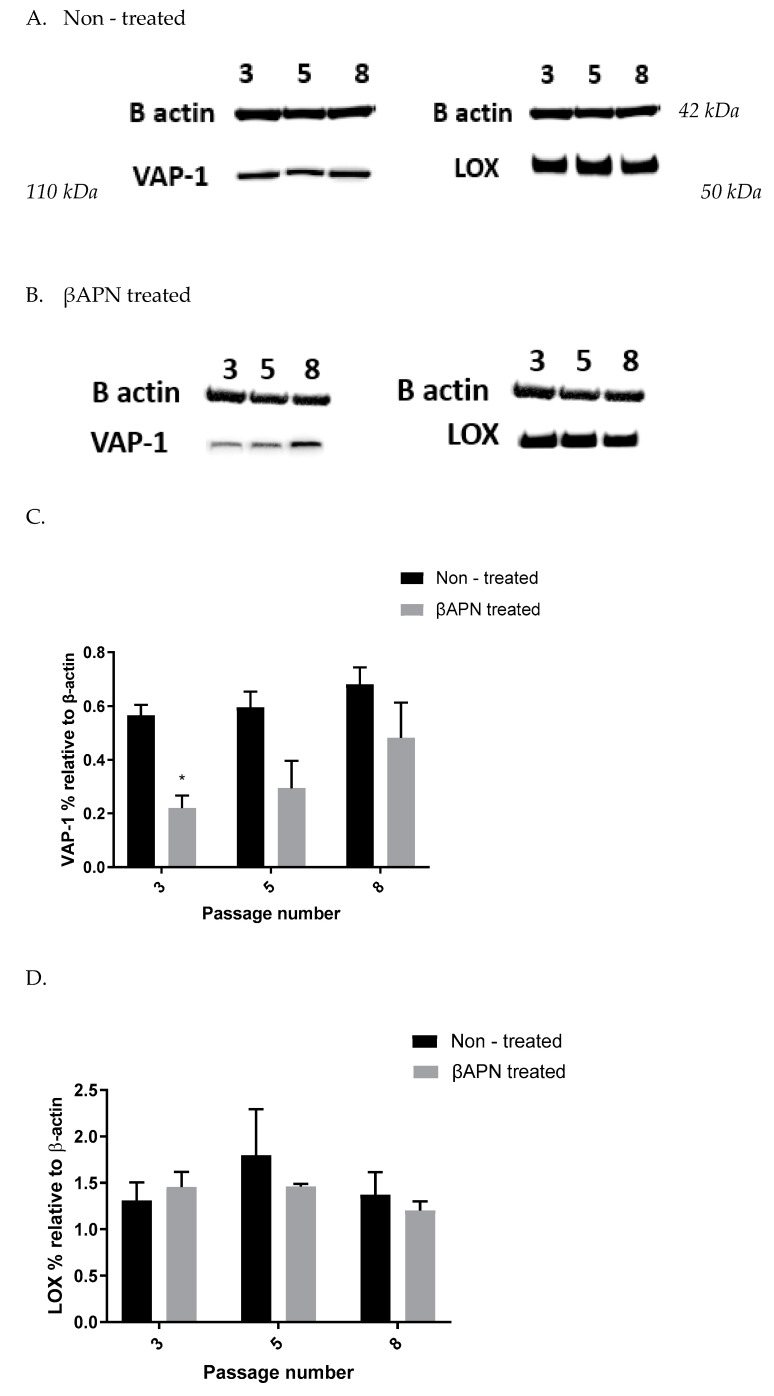
VAP-1 and LOX protein expression in non-treated VSMCs from passages 3, 5, and 8 (**A**) and after 1 h βAPN treatment (**B**). VAP-1 expression (expressed as percentage relative to β-actin) in non-treated (control: black bars) and βAPN treated (grey bars) rat aortic VSMCs from passages 3, 5, and 8 (**C**). There was no significant difference in VAP-1 expression over passage (*p* > 0.05). At passage 3, * *p* < 0.05 for βAPN treated vs. non-treated cells. LOX expression (expressed as percentage relative to β-actin) in non-treated (control: black bars) and βAPN treated (grey bars) rat aortic VSMCs from passages 3, 5, and 8 (**D**). There was no significant difference in LOX expression over passage, or between non-treated vs. βAPN treated cells (*p* > 0.05). Values are mean ± S.E.M. (n = 3).

In order to investigate basal and enzyme-induced changes in VAP-1 and LOX protein expression during VSMCs passaging, cell samples from passages 3, 5, and 8 were lysed without being previously treated (A), or after 1 h treatment with 200 µM βAPN (B). β-actin from each cell sample was detected to normalize the level of protein. The protein was quantified using ImageJ software. Figure 3 shows a representative Western blot of VAP-1 and LOX protein in non-treated cells (A), βAPN treated cells (B), and their quantification graphs (C & D).

### 3.4. VAP-1 and LOX Protein Expression in VSMCs from Passage 3, 5 & 8 at Basal Level and after MDL72527 Treatment

**Figure 4:**
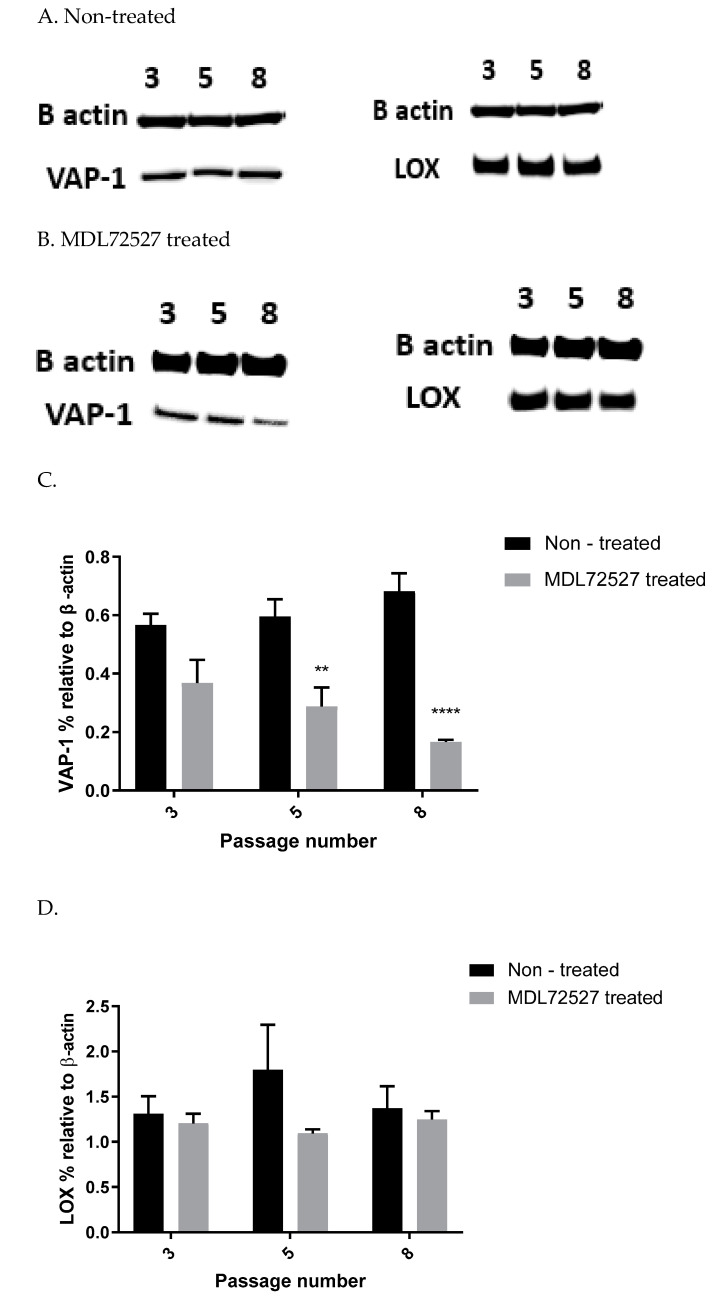
VAP-1 and LOX protein expression in non-treated VSMCs from passages 3, 5, and 8 (**A**) and after 1 h MDL72527 treatment (**B**). VAP-1 expression (expressed as percentage relative to β-actin) in non-treated (control: black bars) and MDL72527 treated (grey bars) rat aortic VSMCs from passages 3, 5 and 8 (**C**). There was no significant difference in VAP-1 expression over passage (*p* > 0.05). At passage 5, (** *p* < 0.01) and passage 8 (**** *p* < 0.0001) for MDL72527 treated vs. non-treated cells. LOX expression (expressed as percentage relative to β-actin) in non-treated (control: black bars) and βAPN treated (grey bars) rat aortic VSMCs from passages 3, 5, and 8 (**D**). There was no significant difference in LOX expression over passage, or between non-treated vs. MDL72527 treated cells (*p* > 0.05). Values are mean ± S.E.M. (n = 3).

In order to investigate basal and enzyme-induced changes in VAP-1 and LOX protein expression during VSMCs passaging, cell samples from passages 3, 5, and 8 were lysed without being previously treated (A), or after 1 h treatment with 100 µM MDL72527 (B). β-actin from each cell sample was detected to normalize the level of protein. The protein was quantified using ImageJ software. Figure 4 shows a representative Western blot of VAP-1 and LOX protein in non-treated cells (A), MDL72527 treated cells (B), and their quantification graphs (C & D).

### 3.5. Aoc3 and LOX mRNA Expression in VSMCs from Passage 3, 5 and 8 after βAPN and MDL72527 Treatment

**Figure 5:**
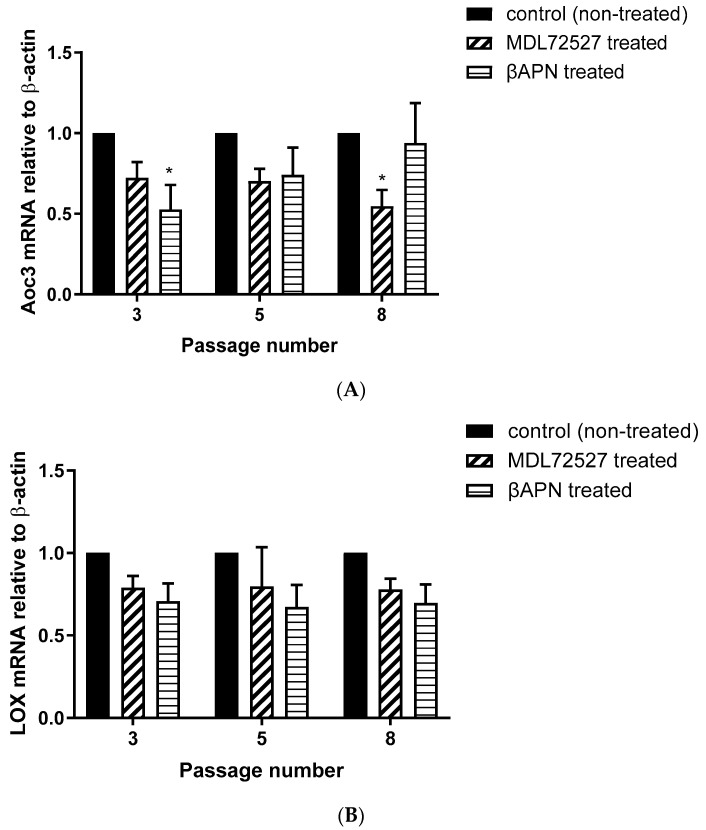
Aoc3 mRNA expression (expressed as fold-change compared with control after normalization to the housekeeping gene β-actin) in non-treated (control: black bars), MDL72527 treated (hatched bars), and βAPN treated (horizontal line bars) VSMCs from passages 3, 5, and 8 (**A**). At passage 3, (* *p* < 0.05 for βAPN treated vs. non-treated cells), and passage 8, (* *p* < 0.05 for MDL72527 treated vs. non-treated cells). LOX mRNA expression (expressed as fold-change compared with control after normalization to the housekeeping gene β-actin) in non-treated (control: black bars), MDL72527 treated (hatched bars) and βAPN treated (horizontal line bars) VSMCs from passages 3, 5, and 8 (**B**). No statistical difference (*p* > 0.05) was detected after βAPN and MDL72527 treatment. Values are mean ± S.E.M. (n = 5).

In order to investigate basal and enzyme-induced changes in Aoc3 and LOX mRNA expression, the RNA was extracted from non-treated and MDL72527 or βAPN treated VSMCs from passages 3, 5, and 8 before performing the quantitative polymerase chain reaction (qPCR). Figure 5A shows the Aoc3 mRNA levels relative to β-actin in non-treated, MDL72527, and βAPN treated cells at passages 3, 5, and 8. Figure 5B shows the LOX mRNA levels relative to β-actin in non-treated, MDL72527, and βAPN treated cells at passages 3, 5, and 8. The 2-ΔΔCT was calculated as the fold-change of the target gene expression in the target sample (LOX expression in MDL72527 and βAPN treated VSMCs from passages 3, 5, and 8 and Aoc3 expression in MDL72527 and βAPN treated VSMCs from passages 3, 5, and 8) relative to the reference sample (LOX expression in non-treated VSMCs from passages 3, 5, and 8 and Aoc3 expression in non-treated VSMCs from passages 3, 5, and 8) and normalized to the reference gene β-actin. The relative gene expression was set to 1 for reference samples, as ΔΔCT is equal to 0.

### 3.6. LOX Knockdown Decrease Aoc3 mRNA and VAP-1 Expression

**Figure 6:**
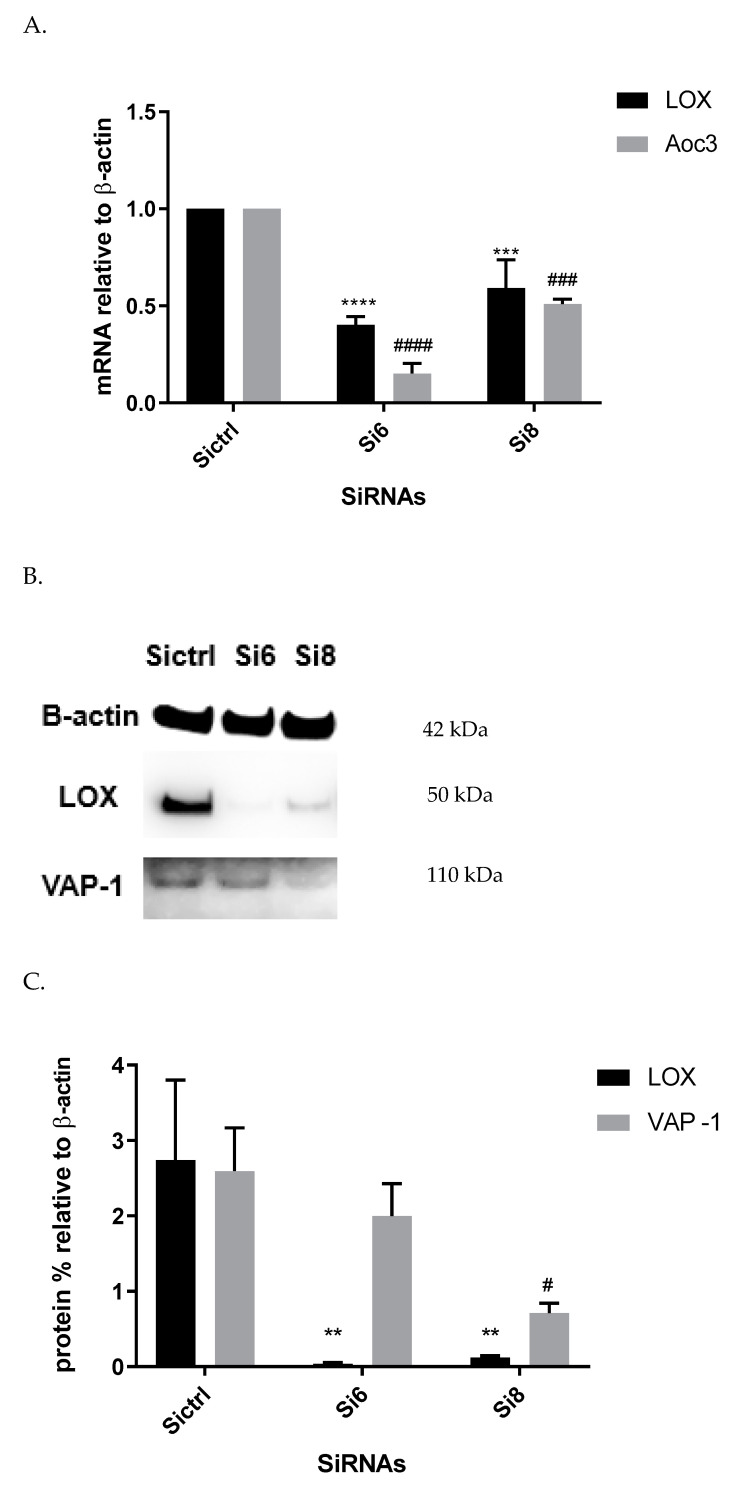
LOX (black bars) and Aoc3 (grey bars) gene expression (expressed as fold-change compared with control after normalization to the housekeeping gene β-actin) in early passage (2 and 3) VSMCs cells treated with Si control, SiRNA6, and SiRNA8 (**A**). For LOX, **** *p* < 0.0001 for Si6 vs. Sictrl and *** *p* < 0.001 for Si8 vs. Sictrl. For Aoc3, ^####^ *p* < 0.0001 for Si6 vs. Sictrl and ^###^ *p* < 0.001 for Si8 vs. Sictrl. LOX and VAP-1 protein expression in rat aortic VSMCs 72 h post transfection with Si control (Sictrl), SiRNA6 (Si6), and SiRNA8 (Si8) (**B**). LOX (black bars) and VAP-1 (grey bars) protein expression (expressed as percentage relative to β-actin) in early passage (2 and 3) VSMCs cells treated with Si control, SiRNA6, and SiRNA8 (**C**). For LOX, ** *p* < 0.01 for Si6 and Si8 vs. Sictrl. For VAP-1, ^#^ *p* < 0.05 for Si8 vs. Sictrl. Values are mean ± S.E.M. (n = 4).

In order to confirm association between SSAO and LOX and to ascertain whether the observed inhibitory effect of βAPN over SSAO activity, protein, and mRNA expression is through direct mechanism, or a consequence of irreversibly inhibited LOX, gene knockdown was performed using two small interfering RNAs (siRNA6 and siRNA8). Figure 6 demonstrates LOX and Aoc3 gene expression after 72 h transfection with siRNA6 and siRNA8. Cells transfected with Si control were used as controls. The 2-ΔΔCT was calculated as the fold-change of the target gene expression in the target sample (Si6 and Si8) relative to the reference sample (Sictrl) and normalized to the reference gene—β-actin. The relative gene expression was set to 1 for reference samples (Sictrl), because ΔΔCT is equal to 0. β-actin from each cell sample was detected to normalize the level of protein. The protein was quantified using ImageJ software.

## 4. Discussion

In order to identify a connection between SSAO and LOX in rat aortic VSMCs, the activity of both enzymes, as well as their protein and mRNA levels, were assessed at the basal level and in the presence of their respective suicide inhibitors MDL72527 and βAPN. In our study, we demonstrated that active LOX regulates SSAO activity, VAP-1 protein, and Aoc3 mRNA expression in early passage rat aortic VSMCs. This effect was first observed from the reduced SSAO activity (expressed as H_2_O_2_ production), VAP-1 expression, and Aoc3 mRNA after βAPN treatment, and was later confirmed by the observed reduction in VAP-1 and Aoc3 mRNA levels after silencing the LOX gene.

Moreover, in our study, we observed a predominant LOX activity in young VSMCs as reflected in the H_2_O_2_ production (Figure 2A,B). Since LOX is the main enzyme which contributes to the maintenance and assembly of the ECM, it is expected to demonstrate predominant activity over SSAO in primary, early passage VSMCs. This is because ECM components are of vital importance in regulating physiological cell processes such as cell proliferation, survival, differentiation, and migration [36]. Our data further demonstrate predominant SSAO activity in cells with a greater passage number (Figure 1A,B). Late passage VSMCs have previously been associated with increased SSAO activity [37,38]. SSAO activity was compared in rat aortic VSMCs from passage 8, 12, 14, and 16 using DMEM with varying glucose concentrations, and a passage dependent increase in SSAO activity was observed in cells grown in high glucose DMEM [37]. In another study, cultured VSMCs were used to study SSAO activity and expression during their differentiation process. The authors of this study have implemented a serum-free medium permissive for in vitro VSMC differentiation, and observed a differentiation-dependent increase in SSAO activity, mRNA, and protein levels [38].

In our data, SSAO activity was predominantly higher in VSMCs, with a greater passage number in comparison to LOX, as reflected in the H_2_O_2_ production (SSAO produced 150 nmol/h/mg protein H_2_O_2_ vs. LOX produced 100 nmol/h/mg protein H_2_O_2_ at VSMCs passage 8) (Figure 1A,B and Figure 2A,B). The higher SSAO activity in late passage VSMCs might have also contributed to the diminished inhibitory potential of βAPN over SSAO. Despite being known as a very selective LOX inhibitor, the effect of βAPN over SSAO has been variably reported in the literature. Depending on the doses, conditions (in vitro or in vivo), and species studied, some studies define βAPN as a reversible competitive inhibitor of SSAO [4]. In our study, at first, βAPN demonstrated inhibitory potential towards SSAO, as it suppressed not only its activity (Figure 1B) but also VAP-1 expression (Figure 3A–C) and Aoc3 mRNA levels (Figure 5A). This inhibition was more pronounced in early passage VSMCs. In cells with a greater passage number, βAPN inhibitory potential over SSAO (activity, protein expression, and mRNA levels) was reduced. Since SSAO has the capacity to regulate the concentration of its substrates by being an ectoenzyme with a catalytically active domain outside the cell surface [39], elevation of its activity in late passage VSMCs might have contributed to an increase in the concentration of its substrate (in this case, benzylamine), which would have consequently resulted in a suppressed inhibitory potential of βAPN. This is because βAPN inhibits SSAO through a competitive reversible mechanism; therefore, increasing the concentration of the substrate would decrease the possibility of the inhibitor binding to the enzyme.

Furthermore, in our study, βAPN demonstrated inhibitory potential over LOX’s activity (Figure 2A); however, it failed to inhibit its protein (Figure 3 A,B,D) and mRNA (Figure 5B). It is important to mention here that in addition to the first characterized LOX, there are four other LOX-like proteins (LOXL 1–4) which are also likely to catalyze a cross-link formation in the vasculature, as does LOX [40], and their degrees of inhibition by βAPN are not yet established. The less potent effect of βAPN over LOX in comparison to SSAO might be attributed to the presence of other LOX active enzymes in these cells. Additionally, it is also known that once the extracellular form of LOX is processed, it can re-enter the cells and localize in the nucleus. This localization is independent of the catalytic activity of the protein and cannot be blocked by a specific LOX inhibitor (βAPN) [41]. Nuclear localizations of LOX have been previously detected within the nuclei of cultured rat aortic smooth muscle cells [41]; therefore, this could be another reason why βAPN was successful in inhibiting LOX activity, but failed to succeed in reducing its protein expression and mRNA levels.

In our study, we observed suppressed SSAO activity (Figure 1 A), VAP-1 expression (Figure 4A–C), and mRNA (Figure 5A) after 1 h MDL72527 treatment. Downregulated SSAO activity has been demonstrated in both lung and aortic tissues after MDL-72274 and MDL72145 treatment, at which SSAO activity was lowered by approximately 70% in aortic tissue and 65% in lung tissue [5]. In our study, MDL72527 was proven to be equally as effective as MDL-72274 and MDL72145 in reducing SSAO activity. Our data demonstrate successful MDL-mediated downregulation of not only SSAO activity, but also VAP-1 expression in primary rat aortic VSMCs with a greater passage number. Reduction in LOX activity has previously been detected with MDL-72274 and MDL-72145 in lung tissue [5]. In light of these findings, our data also demonstrate a reduction in LOX activity after MDL72527 treatment in early passage rat aortic VSMCs (Figure 2B), which could indicate an inhibitory rather than a protein downregulation effect. This is because, as per our data, LOX protein and mRNA failed to be reduced after MDL72527 treatment (Figure 4A,B,D and Figure 5B).

Furthermore, we showed a LOX-driven reduction in SSAO protein and mRNA expression (Figure 6). This indicates that the previously detected βAPN inhibitory potential over SSAO activity and protein expression is through an indirect mechanism, due to irreversibly inhibited LOX. These findings could therefore suggest association between SSAO and LOX in early passage rat aortic VSMCs, where LOX acts as a main regulator of SSAO activity, protein, and mRNA. The interdependent relationship between SSAO and LOX has been associated with physiology and pathology of VSMCs [4,5,14]. It has previously been suggested that SSAO plays a minor role in extracellular matrix stability through its synergistic relationship with LOX [4]. Chronic SSAO inhibition with MDL compounds has led to lesions of disorganization of elastin fibers within tunica media, which was accompanied by degenerative medial changes and metaplastic changes in the VSMCs [5]. LOX, on the other hand, has been shown to oxidize, and, hence, activate membrane-bound cell surface proteins such as VAP-1, which is associated with VAP-1 dependence on LOX [14], as is reflected in our data (Figure 3A–C). Downregulated SSAO activity, protein, and mRNA expression due to downregulated LOX (as observed in our study) could accelerate further damage to the ECM stability, impairing vascular tone regulation and, consequently, posing a greater risk for the development of atherosclerosis. Further studies to address the knockdown of the Aoc3 gene and observe any effect on LOX activity, protein, and mRNA expression would highlight whether the MDL72527 effect on LOX activity is inhibitory (as observed in our study) or whether it is a consequence of irreversibly inhibited SSAO.

## 5. Conclusions

Our data show predominant LOX activity in early passage and predominant SSAO activity in late passage rat aortic VSMCs. It also demonstrates association between these two enzymes in early passage rat aortic VSMCs, where LOX was identified as a regulator of SSAO activity, VAP-1 protein, and Aoc3 mRNA expression. Additional studies are needed to further investigate the relationship between LOX and SSAO in rat aortic VSMCs, and to demonstrate how this might contribute to the development of atherosclerosis.

## Figures and Tables

**Table 1:** Primers sequence for Rattus norvegicus amine oxidase, copper containing 3 (Aoc3), Rattus norvegicus lysyl oxidase (LOX), and Rattus norvegicus β-actin.

*Gene Name*	*Forward Primer (5′–3′)*	*Reverse Primer (5′–3′)*
*Aoc3*	ACCCACAACGCTCACTTCA	TTCATAGGGACAAAAGCCAAA
*LOX*	AGGATCCACGGAGGATGG	GGGAGGCCAGGAGACACT
*β-actin*	CCCGCGAGTACAACCTTCT	CGTCATCCATGGCGAACT

**Table 2:** An illustration of the qPCR experimental design.

	*Reference Sample*	*Target Sample*
*Reference gene*	β-actin expression in non-treated VSMCs from passage 3, 5 and 8	β-actin expression in MDL72527 and βAPN treated VSMCs from passage 3, 5 and 8
*Target gene 1*	*Aoc3* expression in non-treated VSMCs from passage 3, 5 and 8	*Aoc3* expression in MDL72527 and βAPN treated VSMCs from passage 3, 5 and 8
*Target gene 2*	*LOX* expression in non-treated VSMCs from passage 3, 5 and 8	*LOX* expression in MDL72527 and βAPN treated VSMCs from passage 3, 5 and 8

**Table 3:** LOX siRNAs and their target sequences.

*SiRNA*	*Target Sequence*
*SiRNA_LOX_6*	AGGGCGGATGTCAGAGACTAT
*SiRNA_LOX_8*	TCCCGGATGTTATGATACTTA

**Figure A1:**
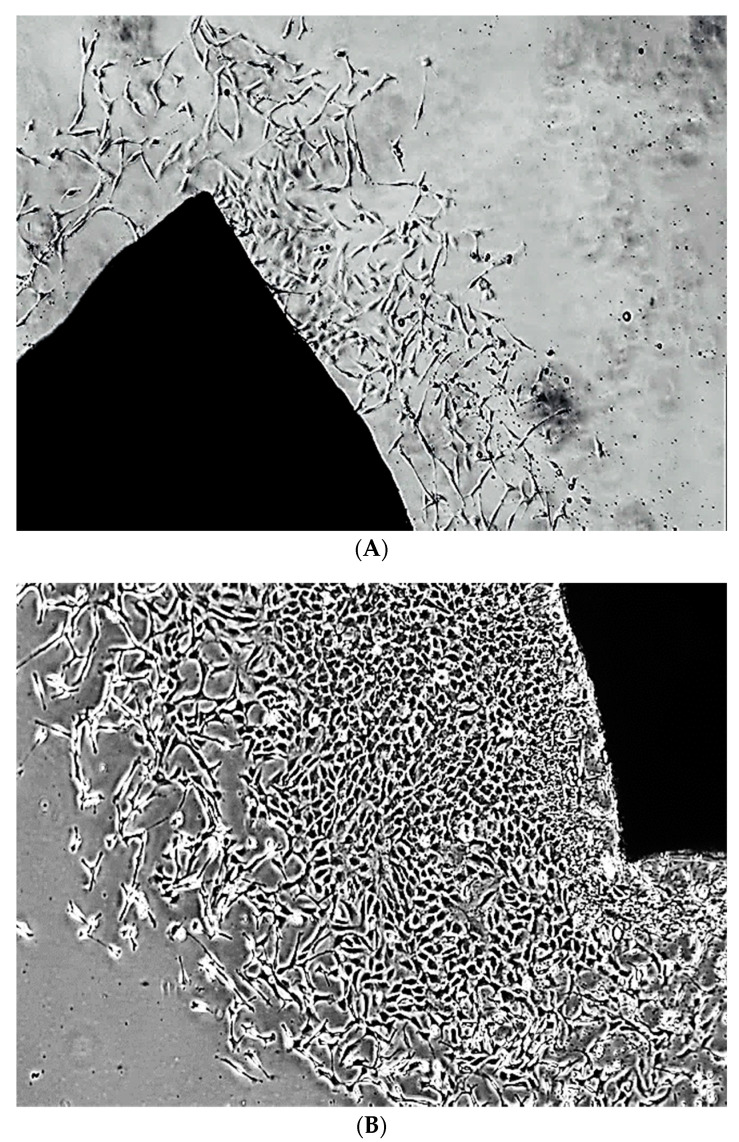
In vitro culture of rat aortic VSMCs from aortic explants viewed at 160 X magnification (10 X E-PL, 4 X objective lens, 40 × image magnification) under an inverted Olympus light microscope. (**A**) Cell growth after 4 days incubation; (**B**) cell growth after 7 days incubation (scale bar: 27.2851 μm).

**Figure A2:**
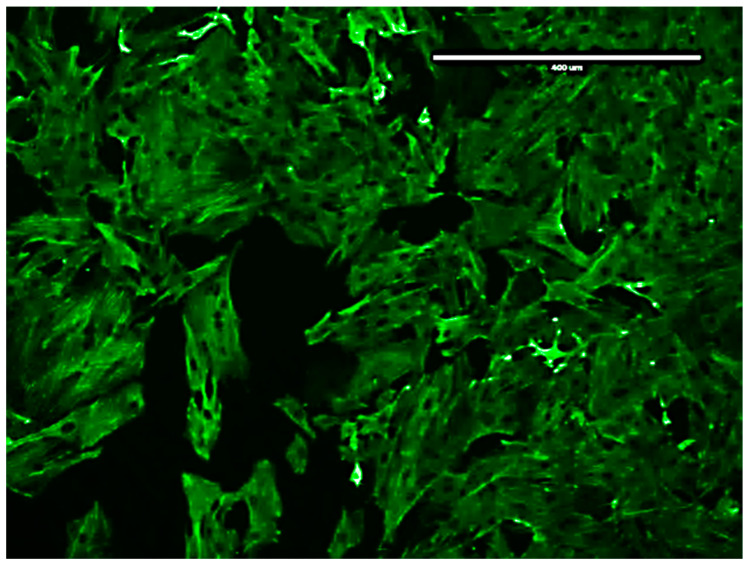
Immuno-stained microscopic image of rat aortic VSMCs depicting the presence of SM22α actin fibers (scale bar: 400 μm, magnification: × 10).

## Data Availability

The data presented in this study are available on request from the corresponding author. The data are not publicly available due to privacy concerns.

## Appendix A

Figure A1 shows an early micrograph of the VSMCs beginning to migrate from the explant after 4 (A) and 7 (B) days incubation.

Figure A2 is a visual presentation of the stained rat aortic VSMCs depicting the presence of SM22α actin fibers.
